# Supplementary material for: Modeling glioblastoma heterogeneity as a dynamic network of cell states
Source: Mol Syst Biol. 2021 Sep 16;17(9):e10105. doi: 10.15252/msb.202010105 (PMC8444284; doi:10.15252/msb.202010105)
Supplement: Supplementary file 6 — Source Data for Figure 5 [file MSB-17-e10105-s004.zip › Figure5A_sourcedata/GSEA_3017/nosigdb_stateE.GseaPreranked.1622795031093/DALMO_BARCODENUMBER_UNPUB.html]

Details for gene set DALMO\_BARCODENUMBER\_UNPUB[GSEA]

|  || Dataset | state63017 |
| Phenotype | NoPhenotypeAvailable |
| Upregulated in class | na\_neg |
| GeneSet | DALMO\_BARCODENUMBER\_UNPUB |
| Enrichment Score (ES) | -0.7203259 |
| Normalized Enrichment Score (NES) | -3.9297884 |
| Nominal p-value | 0.0 |
| FDR q-value | 0.0 |
| FWER p-Value | 0.0 |
Table: GSEA Results Summary

  

Fig 1: Enrichment plot: DALMO\_BARCODENUMBER\_UNPUB      
 Profile of the Running ES Score & Positions of GeneSet Members on the Rank Ordered List

  

| PROBE | GENE SYMBOL | GENE\_TITLE | RANK IN GENE LIST | RANK METRIC SCORE | RUNNING ES | CORE ENRICHMENT || 1 | SPARCL1 |  |  | 7 | 0.963 | 0.0040 | No |
| 2 | GPC1 |  |  | 50 | 0.530 | -0.0392 | No |
| 3 | S100A10 |  |  | 51 | 0.527 | -0.0325 | No |
| 4 | MMP2 |  |  | 57 | 0.503 | -0.0320 | No |
| 5 | BEX1 |  |  | 88 | 0.419 | -0.0624 | No |
| 6 | LONRF2 |  |  | 118 | 0.365 | -0.0922 | No |
| 7 | PCDH9 |  |  | 121 | 0.359 | -0.0900 | No |
| 8 | S100A6 |  |  | 176 | 0.312 | -0.1503 | No |
| 9 | DKK3 |  |  | 205 | 0.293 | -0.1799 | No |
| 10 | GSTM3 |  |  | 257 | 0.259 | -0.2373 | No |
| 11 | CHPF |  |  | 269 | 0.251 | -0.2472 | No |
| 12 | DAZAP1 |  |  | 299 | -0.258 | -0.2784 | No |
| 13 | HMGA1 |  |  | 313 | -0.261 | -0.2905 | No |
| 14 | PAICS |  |  | 336 | -0.265 | -0.3133 | No |
| 15 | PLK4 |  |  | 551 | -0.334 | -0.5638 | No |
| 16 | TROAP |  |  | 562 | -0.341 | -0.5714 | No |
| 17 | LSM5 |  |  | 568 | -0.342 | -0.5729 | No |
| 18 | GDF15 |  |  | 575 | -0.347 | -0.5757 | No |
| 19 | HMGB3 |  |  | 577 | -0.348 | -0.5724 | No |
| 20 | POC1A |  |  | 611 | -0.363 | -0.6070 | No |
| 21 | KIF18B |  |  | 612 | -0.363 | -0.6024 | No |
| 22 | SEZ6L |  |  | 642 | -0.383 | -0.6320 | No |
| 23 | PHF19 |  |  | 648 | -0.391 | -0.6330 | No |
| 24 | H2AFV |  |  | 684 | -0.423 | -0.6692 | No |
| 25 | DIAPH3 |  |  | 696 | -0.427 | -0.6769 | No |
| 26 | ANP32B |  |  | 699 | -0.431 | -0.6737 | No |
| 27 | PARPBP |  |  | 705 | -0.434 | -0.6742 | No |
| 28 | MZT1 |  |  | 711 | -0.437 | -0.6745 | No |
| 29 | TK1 |  |  | 747 | -0.480 | -0.7100 | No |
| 30 | PRR11 |  |  | 755 | -0.488 | -0.7121 | No |
| 31 | SPDL1 |  |  | 758 | -0.491 | -0.7082 | No |
| 32 | CDCA3 |  |  | 759 | -0.497 | -0.7019 | No |
| 33 | LRR1 |  |  | 768 | -0.512 | -0.7048 | No |
| 34 | CENPA |  |  | 782 | -0.538 | -0.7134 | Yes |
| 35 | SKA3 |  |  | 783 | -0.539 | -0.7065 | Yes |
| 36 | FANCD2 |  |  | 787 | -0.544 | -0.7032 | Yes |
| 37 | CDC20 |  |  | 793 | -0.562 | -0.7019 | Yes |
| 38 | HMGN2 |  |  | 795 | -0.564 | -0.6959 | Yes |
| 39 | RACGAP1 |  |  | 796 | -0.566 | -0.6887 | Yes |
| 40 | HMGB1 |  |  | 797 | -0.567 | -0.6814 | Yes |
| 41 | TUBB4B |  |  | 804 | -0.573 | -0.6812 | Yes |
| 42 | CENPN |  |  | 805 | -0.574 | -0.6739 | Yes |
| 43 | PLK1 |  |  | 809 | -0.586 | -0.6700 | Yes |
| 44 | DEK |  |  | 812 | -0.591 | -0.6648 | Yes |
| 45 | FOXM1 |  |  | 814 | -0.593 | -0.6584 | Yes |
| 46 | CDCA2 |  |  | 816 | -0.597 | -0.6519 | Yes |
| 47 | PIF1 |  |  | 822 | -0.616 | -0.6500 | Yes |
| 48 | CKAP2L |  |  | 826 | -0.625 | -0.6456 | Yes |
| 49 | AURKB |  |  | 829 | -0.634 | -0.6399 | Yes |
| 50 | PIMREG |  |  | 833 | -0.641 | -0.6353 | Yes |
| 51 | CENPW |  |  | 835 | -0.643 | -0.6282 | Yes |
| 52 | CDKN3 |  |  | 836 | -0.653 | -0.6199 | Yes |
| 53 | BUB1 |  |  | 837 | -0.653 | -0.6115 | Yes |
| 54 | ANP32E |  |  | 841 | -0.676 | -0.6064 | Yes |
| 55 | BIRC5 |  |  | 843 | -0.679 | -0.5989 | Yes |
| 56 | CENPM |  |  | 844 | -0.682 | -0.5902 | Yes |
| 57 | NCAPH |  |  | 845 | -0.685 | -0.5815 | Yes |
| 58 | SPC24 |  |  | 848 | -0.694 | -0.5750 | Yes |
| 59 | DHFR |  |  | 851 | -0.701 | -0.5684 | Yes |
| 60 | CIP2A |  |  | 853 | -0.712 | -0.5605 | Yes |
| 61 | KIF2C |  |  | 855 | -0.721 | -0.5524 | Yes |
| 62 | NUF2 |  |  | 856 | -0.721 | -0.5432 | Yes |
| 63 | ANLN |  |  | 860 | -0.733 | -0.5374 | Yes |
| 64 | KIF4A |  |  | 861 | -0.736 | -0.5280 | Yes |
| 65 | CCNB2 |  |  | 865 | -0.745 | -0.5220 | Yes |
| 66 | ARHGAP11A |  |  | 867 | -0.756 | -0.5136 | Yes |
| 67 | CCNA2 |  |  | 872 | -0.770 | -0.5085 | Yes |
| 68 | KIF23 |  |  | 873 | -0.772 | -0.4986 | Yes |
| 69 | NCAPG2 |  |  | 879 | -0.781 | -0.4946 | Yes |
| 70 | PTTG1 |  |  | 880 | -0.791 | -0.4844 | Yes |
| 71 | H2AFX |  |  | 882 | -0.799 | -0.4754 | Yes |
| 72 | KIFC1 |  |  | 883 | -0.807 | -0.4651 | Yes |
| 73 | SGO1 |  |  | 889 | -0.822 | -0.4605 | Yes |
| 74 | KIF15 |  |  | 890 | -0.834 | -0.4499 | Yes |
| 75 | CDCA5 |  |  | 892 | -0.841 | -0.4403 | Yes |
| 76 | H2AFZ |  |  | 893 | -0.849 | -0.4294 | Yes |
| 77 | KPNA2 |  |  | 895 | -0.858 | -0.4196 | Yes |
| 78 | SMC2 |  |  | 897 | -0.867 | -0.4097 | Yes |
| 79 | SGO2 |  |  | 898 | -0.869 | -0.3986 | Yes |
| 80 | CENPE |  |  | 899 | -0.873 | -0.3875 | Yes |
| 81 | PBK |  |  | 900 | -0.880 | -0.3762 | Yes |
| 82 | MIS18BP1 |  |  | 904 | -0.889 | -0.3684 | Yes |
| 83 | CCNB1 |  |  | 906 | -0.895 | -0.3582 | Yes |
| 84 | CKS1B |  |  | 909 | -0.912 | -0.3489 | Yes |
| 85 | HJURP |  |  | 910 | -0.921 | -0.3371 | Yes |
| 86 | CKS2 |  |  | 911 | -0.922 | -0.3253 | Yes |
| 87 | GTSE1 |  |  | 912 | -0.941 | -0.3133 | Yes |
| 88 | KIF11 |  |  | 913 | -0.957 | -0.3010 | Yes |
| 89 | MAD2L1 |  |  | 921 | -0.990 | -0.2967 | Yes |
| 90 | UBE2T |  |  | 922 | -0.994 | -0.2840 | Yes |
| 91 | DLGAP5 |  |  | 923 | -0.998 | -0.2712 | Yes |
| 92 | SPC25 |  |  | 924 | -1.033 | -0.2580 | Yes |
| 93 | ESCO2 |  |  | 926 | -1.067 | -0.2455 | Yes |
| 94 | UBE2C |  |  | 928 | -1.108 | -0.2325 | Yes |
| 95 | NUSAP1 |  |  | 929 | -1.112 | -0.2183 | Yes |
| 96 | TPX2 |  |  | 930 | -1.124 | -0.2040 | Yes |
| 97 | NCAPG |  |  | 931 | -1.142 | -0.1893 | Yes |
| 98 | ASPM |  |  | 932 | -1.146 | -0.1747 | Yes |
| 99 | CENPF |  |  | 935 | -1.200 | -0.1617 | Yes |
| 100 | HMGB2 |  |  | 937 | -1.238 | -0.1471 | Yes |
| 101 | CDK1 |  |  | 938 | -1.248 | -0.1311 | Yes |
| 102 | RRM2 |  |  | 940 | -1.403 | -0.1144 | Yes |
| 103 | MKI67 |  |  | 941 | -1.407 | -0.0964 | Yes |
| 104 | TOP2A |  |  | 942 | -1.417 | -0.0782 | Yes |
| 105 | PCLAF |  |  | 943 | -1.554 | -0.0584 | Yes |
| 106 | HIST1H1B |  |  | 945 | -1.903 | -0.0352 | Yes |
| 107 | HIST1H4C |  |  | 946 | -2.752 | 0.0000 | Yes |
Table: GSEA details [plain text format]

  

Fig 2: DALMO\_BARCODENUMBER\_UNPUB: Random ES distribution      
 Gene set null distribution of ES for **DALMO\_BARCODENUMBER\_UNPUB**
